# Role of Magnesium in Diabetic Nephropathy for Better Outcomes

**DOI:** 10.7759/cureus.43076

**Published:** 2023-08-07

**Authors:** Mahesh Mamilla, Sai Goutham Reddy Yartha, Richa Tuli, Sunil Konipineni, Dharma Teja Rayaprolu, Gargi Borgharkar, Pavan Kumar Reddy Kalluru, Thanmai Reddy Thugu

**Affiliations:** 1 Internal Medicine, Sri Venkateswara Medical College, Tirupati, IND; 2 Internal Medicine, School of Medicine, Xiamen University, Xiamen, CHN; 3 Internal Medicine, Zaporizhzhia State Medical University, Zaporizhzhia, UKR; 4 Internal Medicine, Mamata Medical College, Khammam, IND; 5 Public Health, University of Alabama at Birmingham, Birmingham, USA; 6 Internal Medicine, Sri Padmavathi Medical College for Women, Sri Venkateswara Institute of Medical Sciences, Tirupati, IND

**Keywords:** progression of ckd, end-stage renal disease (esrd), anti-inflammatory effect, diabetic nephropathy (dn), magnesium levels

## Abstract

Diabetic nephropathy (DN) is a major cause of end-stage renal disease worldwide, resulting from uncontrolled diabetes. Oxidative stress plays a critical role in the pathophysiology of DN, leading to cellular damage and disease progression. Magnesium, an essential mineral, has emerged as a potential therapeutic agent due to its antioxidative, anti-inflammatory, and antifibrotic properties. An extensive literature search was conducted on Medline using the keywords “Diabetic nephropathy,” “Magnesium,” and “Chronic Kidney Disease,” and the results published after 2000 were exclusively studied to build this review. This review aims to summarize the role of magnesium in DN and explore its therapeutic potential. Magnesium acts as a cofactor for antioxidant enzymes, directly scavenges reactive oxygen species, and enhances the expression of antioxidant proteins. Furthermore, magnesium exhibits anti-inflammatory effects by suppressing pro-inflammatory cytokine production and inhibiting inflammatory signaling pathways. Magnesium supplementation has been shown to reduce oxidative stress markers and improve antioxidant enzyme activities in clinical studies. Additionally, magnesium has been found to mitigate renal fibrosis, maintain tubular integrity and function, improve endothelial function, and modulate renal hemodynamics. Although limited clinical trials suggest the renoprotective effects of magnesium in DN, further research is needed to determine the optimal dosage, duration, and long-term effects of magnesium supplementation. Despite existing drawbacks and gaps in the literature, magnesium holds promise as adjunctive therapy for DN by targeting oxidative stress and preserving renal function.

## Introduction and background

Diabetic nephropathy (DN) is a progressive kidney disease and a serious consequence of uncontrolled diabetes that continues to be a significant cause of end-stage renal disease (ESRD) globally. DN is diagnosed if persistent albuminuria (>300 mg/day or >200 μg/minute) is confirmed on at least two occasions within three to six months, along with a progressive decline in the glomerular filtration rate [1]. The pathophysiology of DN is complex, involving various processes, such as oxidative stress, inflammation, and altered renal hemodynamics, with persistent hyperglycemia being a major driving force. Among the complexities of DN pathophysiology, oxidative stress has emerged as a key mediator, producing cellular damage and contributing to disease development [2-4].

Studies have been investigating potential therapeutic ways to slow the progression of DN and improve patient outcomes for many years [5]. Magnesium, an important mineral involved in many physiological processes, has attracted considerable attention recently because of its potential protective function in the development and progression of DN [6]. Magnesium supplementation has been linked to improved outcomes in animal and clinical research for the treatment of diabetes and its complications [7,8]. Understanding the molecular foundations of magnesium in DN and researching its therapeutic implications is critical for developing fresh strategies to treat this devastating illness.

This review aims to summarize the current knowledge on the role of magnesium in DN and shed light on its potential as a treatment strategy to reduce oxidative stress damage and preserve renal function in diabetic patients. In addition, the review explores how magnesium exerts its renoprotective effects, including its antioxidant, anti-inflammatory, and antifibrotic capabilities. Finally, it discusses the drawbacks and potential gaps of using magnesium in DN. We can potentially pave the road for more effective and personalized treatments for DN patients if we address these information gaps.

## Review

Diabetic nephropathy

Both people with type 1 and type 2 diabetes are susceptible to DN, a progressive kidney condition caused by poorly treated diabetes mellitus. Surprisingly, DN is one of the main causes of ESRD globally. DN is diagnosed when there is consistent albuminuria (>300 mg/day or >200 μg/minute) on two separate occasions, with a gap of three to six months between the tests, along with a gradual decrease in the glomerular filtration rate [1]. A complex combination of metabolic, hemodynamic, and inflammatory variables underlies the pathogenesis of DN [9]. Persistent hyperglycemia, which causes the buildup of advanced glycation end products (AGEs) in renal tissues, is a critical component in the development of DN [10]. The AGE receptor (RAGE) is one of the physiological pathways that is activated by the presence of AGEs to cause oxidative stress and inflammation [11]. Reactive oxygen species (ROS) are produced as a result of increased oxidative stress, which causes cellular damage and contributes to kidney fibrosis [12]. This complicated chain of events emphasizes the severity of DN and its impact on renal function in diabetics.

Aside from oxidative stress, the renin-angiotensin-aldosterone system (RAAS) is important in the development of DN. Chronic hyperglycemia and hemodynamic alterations promote angiotensin II production, a strong vasoconstrictor and pro-inflammatory mediator [13]. Angiotensin II causes renal inflammation, fibrosis, and apoptosis via various mechanisms, including the overexpression of transforming growth factor-beta (TGF-β) and the synthesis of connective tissue growth factor (CTGF), all of which promote fibrosis [14,15]. Renal hemodynamic changes, such as glomerular hyperfiltration and increased intraglomerular pressure, aggravate the course of DN. The combination of hyperglycemia-induced efferent arteriolar dilatation and increased angiotensin II levels causes glomerular hypertension and hyperfiltration, resulting in glomerular basement membrane degradation and podocyte injury [16,17]. As a result, podocyte dysfunction affects the integrity of the glomerular filtration barrier, resulting in albuminuria, which is characterized by protein leakage into the urine [18]. The complex interplay of chronic hyperglycemia, RAAS activation, and hemodynamic alterations greatly contributes to the development and progression of DN, highlighting the multifaceted nature of this kidney disease in diabetics.

**Figure 1:**
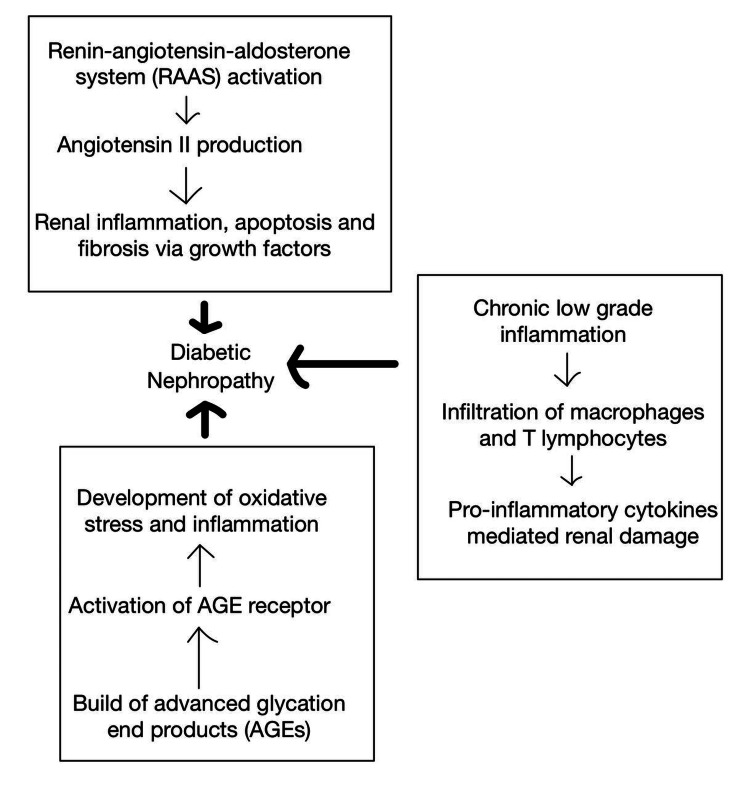
Metabolic, hemodynamic, and inflammatory variables underlying the pathogenesis of diabetic nephropathy. The image is the authors’ own creation.

Furthermore, chronic low-grade inflammation contributes significantly to the development of DN. The infiltration of immune cells into renal tissues, such as macrophages and T lymphocytes, causes the release of pro-inflammatory cytokines, such as tumor necrosis factor-alpha (TNF-α) and interleukin-6 (IL-6) [19]. These cytokines activate inflammatory signaling pathways, which cause endothelial dysfunction, fibrosis, and tubulointerstitial damage, further worsening the course of DN [20]. Importantly, various risk factors might hasten the progression of DN, increasing its complexity. Hypertension, dyslipidemia, obesity, smoking, and familial predisposition are among the factors that can contribute to the exacerbation of the pathophysiological processes of DN [21]. Understanding the role of chronic low-grade inflammation and the impact of these risk factors is critical for developing effective prevention methods to manage and mitigate the impact of DN on diabetes patients. The metabolic, hemodynamic, and inflammatory variables discussed above in the pathogenesis of DN are represented in Figure 1.

Role of Oxidative Stress

**Figure 2:**
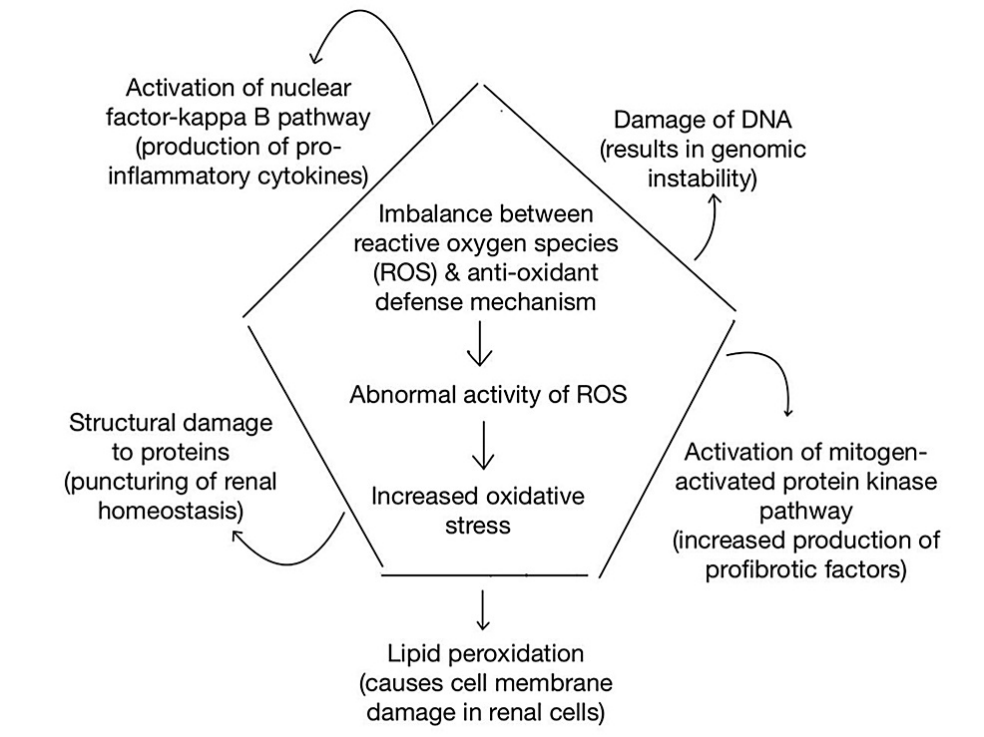
Effects of oxidative stress. The image is the authors’ own creation.

The pathophysiology of DN, a progressive kidney disease that arises as a consequence of diabetes mellitus, is heavily influenced by oxidative stress. An imbalance between the generation of ROS and the antioxidant defense system causes oxidative stress, which causes cellular damage and contributes to the development and progression of DN [22], as represented in Figure 2. Persistent hyperglycemia is a primary cause of oxidative stress in the kidneys of diabetics. High glucose levels boost ROS production by various mechanisms, including increased glucose metabolism via the polyol pathway, activation of protein kinase C, and increased mitochondrial electron transport chain activity [23,24]. These processes result in excess superoxide anions and other ROS, which overwhelms the antioxidant defense mechanisms.

ROS can harm a variety of biological components, including lipids, proteins, and nucleic acids. Lipid peroxidation is a prominent consequence of ROS activity that causes oxidative damage to cell membranes, resulting in structural and functional degeneration of renal cells [25]. Furthermore, ROS-induced protein oxidation and nitration affect protein structure and function, influencing critical cellular processes involved in maintaining renal homeostasis [26]. Moreover, oxidative stress-induced DNA damage can lead to genomic instability, accelerating the course of DN [27]. Furthermore, oxidative stress activates numerous signaling pathways that are involved in the pathogenesis of DN. The nuclear factor-kappa B (NF-κB) pathway is one such mechanism that is activated by ROS. As a result, it stimulates the production of pro-inflammatory cytokines, such as TNF-α and IL-6, resulting in kidney inflammation and injury [28]. Furthermore, oxidative stress activates the mitogen-activated protein kinase signaling pathway, resulting in an increase in profibrotic factors such as TGF-β and CTGF. These variables play a role in the development of renal fibrosis in DN patients [29,30]. Other diabetes-related variables, other than hyperglycemia, also contribute to oxidative stress in DN. Dyslipidemia, defined by increased triglycerides and low-density lipoproteins (LDLs), exacerbates ROS formation while decreasing antioxidant defenses [31]. Furthermore, AGEs, which are generated by the non-enzymatic interaction of glucose with proteins, increase oxidative stress. AGEs increase the generation of ROS and activate inflammatory pathways [32]. Overall, oxidative stress and the ensuing activation of numerous signaling pathways play an important role in the development of DN, with variables such as dyslipidemia and AGEs also contributing.

Oxidative stress serves a dual role in DN, contributing to its initiation while also acting as a critical mediator of its downstream effects. Renal cells, including podocytes, endothelial cells, and tubular cells, are damaged by oxidative stress, resulting in malfunction and cell death, which contributes to renal function loss in DN [17,33]. Furthermore, oxidative stress contributes to the development of albuminuria, a defining hallmark of DN, by weakening the glomerular filtration barrier [34]. Recognizing the importance of oxidative stress in DN, promising outcomes from preclinical and clinical treatments targeting oxidative stress pathways have emerged. N-acetylcysteine, magnesium, vitamin E, and alpha-lipoic acid have been shown to have renoprotective benefits in experimental DN models. They accomplish this by lowering oxidative stress and enhancing renal function.

Role of magnesium

Magnesium, an essential mineral with various physiological functions, has been recognized for its potential role in reducing oxidative stress damage. Magnesium’s ability to mitigate oxidative stress is attributed to its involvement in multiple cellular and enzymatic processes that regulate antioxidant defense mechanisms [35]. One of the mechanisms by which magnesium reduces oxidative stress is its direct antioxidant properties. Magnesium acts as a cofactor for several antioxidant enzymes, including superoxide dismutase (SOD), glutathione peroxidase (GPx), and catalase. These enzymes play a crucial role in neutralizing ROS and preventing cellular damage [35,36]. Magnesium enhances the activity of SOD, which converts superoxide radicals into less reactive species, thereby reducing oxidative stress [37]. Furthermore, magnesium supports the function of GPx, which uses glutathione to detoxify hydrogen peroxide and lipid hydroperoxides, thereby preventing oxidative damage [38]. The presence of adequate magnesium levels is essential for optimal enzymatic activity and efficient ROS scavenging.

In addition to its direct antioxidant actions, magnesium influences the expression and activity of several proteins involved in the regulation of oxidative stress. Magnesium has been demonstrated to increase the expression of the transcription factor nuclear factor erythroid 2-related factor 2 (Nrf2), which is important for activating the antioxidant response element (ARE) pathway [39]. When the Nrf2-ARE pathway is activated, the synthesis of endogenous antioxidants such as glutathione and heme oxygenase-1 (HO-1) increases, which protects against oxidative stress-induced cellular damage [40]. The activation of Nrf2 by magnesium helps to decrease oxidative stress and enhances cellular resilience.

Furthermore, magnesium exhibits anti-inflammatory properties, which indirectly contribute to the reduction of oxidative stress damage. Inflammation and oxidative stress are interconnected processes, with oxidative stress triggering and perpetuating inflammatory responses. Magnesium has been shown to suppress the production of pro-inflammatory cytokines, such as IL-6 and TNF-α, and inhibit the activation of NF-κB, a key transcription factor involved in the inflammatory response [7,41]. By attenuating inflammation, magnesium indirectly reduces the generation of ROS and oxidative stress damage. Several studies have examined the effects of magnesium supplementation on reducing oxidative stress in various clinical diseases. Magnesium supplementation has been demonstrated to reduce oxidative stress markers such as malondialdehyde and improve antioxidant enzyme activities such as SOD and GPx in diabetic individuals [19,42]. Similarly, magnesium supplementation has been shown to have antioxidative effects in people with cardiovascular disease by lowering lipid peroxidation and enhancing antioxidant capacity [43,44].

In another study, magnesium supplementation enhanced redox balance and decreased oxidative stress in hemodialysis patients [8]. These data point to magnesium’s potential as a therapeutic strategy for lowering oxidative stress-related damage in chronic kidney disease (CKD). Furthermore, magnesium has antifibrotic properties in CKD. Renal fibrosis, defined by an excess of extracellular matrix components, is a characteristic of CKD development. Magnesium supplementation has been found to reduce renal fibrosis by suppressing the production of profibrotic factors such as TGF-β and CTGF [45,46]. Magnesium’s capacity to alter the TGF-β signaling pathway and downstream profibrotic mediators helps to prevent or reduce renal fibrosis, preserving renal structure and function. Furthermore, it has been found that magnesium helps maintain tubular integrity and function. Tubular damage is a prevalent characteristic of CKD and can contribute to decreased renal function. Magnesium supplementation has been demonstrated to increase tubular cell survival and integrity, sustaining tubular function in patients with CKD [47]. The effects of magnesium on tubular cells may include the modulation of cell signaling pathways as well as the regulation of cellular transporters involved in tubular reabsorption and secretion. Magnesium maintains renal homeostasis and contributes to the preservation of renal function by maintaining tubular integrity and function [47].

Moreover, magnesium has been shown to have vasoprotective benefits in CKD patients. CKD is characterized by endothelial dysfunction and vascular calcification, both of which lead to cardiovascular problems. Magnesium supplementation has been found to improve endothelial function by increasing the generation of nitric oxide (NO) and decreasing vascular calcification [48,49]. Magnesium’s capacity to increase endothelial function and prevent arterial calcification contributes to vascular health preservation and may lower the risk of cardiovascular events in CKD patients. In the bargain, magnesium has been linked to the modulation of renal hemodynamics. Renal damage in CKD is exacerbated by changes in renal hemodynamics, such as glomerular hyperfiltration and increased intraglomerular pressure. Magnesium supplementation has been demonstrated to lower intraglomerular pressure and attenuate glomerular hyperfiltration, thus protecting against renal damage [45,46]. These effects can be mediated by modulating renal vasoactive factors and improving renal autoregulation. Magnesium helps to maintain renal function and minimize renal damage in CKD by modulating renal hemodynamics.

As an additional token, clinical investigations have found that magnesium supplementation has renoprotective effects in CKD patients. Magnesium supplementation enhanced renal function and decreased urine protein excretion in CKD patients in a randomized controlled experiment [48]. In another investigation, magnesium supplementation reduced renal interstitial fibrosis and improved kidney histological abnormalities in a CKD animal model [35]. These findings show magnesium’s potential as an additional therapy for slowing the course of CKD and preserving renal function.

Apart from the above-mentioned mechanisms, magnesium has other effects which show renal protection. Very limited studies are available in the literature, and further studies are needed for a concrete opinion. The effect of magnesium on blood pressure and endothelial function is notable. Higher serum magnesium levels in CKD patients have been linked to better endothelial dysfunction, as seen by greater flow-mediated dilation of the brachial artery [50]. Higher serum magnesium levels, on the other hand, have been linked to a decreased risk of developing hypertension in individuals who do not have CKD [51]. The vasodilatory characteristics of magnesium, as well as its potential to improve endothelial function, play important roles in these connections. Magnesium functions as a natural calcium channel blocker, increasing vasodilation and decreasing blood vessel constriction. It also influences the activity of endothelial nitric oxide synthase, resulting in increased NO generation and improved endothelial function [51]. One study conducted in 2023 focused on the relationship between DN, gut microbiota, and magnesium. The study suggested that magnesium lithospermate B can ameliorate DN by inhibiting the formation of uremic toxins, which are known to contribute to kidney damage. The mechanism of action involves the modulation of gut microbiota [52]. However, as mentioned above, further extensive studies are required to reach a definite conclusion.

Drawbacks and potential gaps

The literature on using magnesium as a therapy for DN has notable gaps. Limited research trials with small sample sizes have explored magnesium as a potential treatment option. All studies mentioned above focused on the relationship between magnesium and DN. To establish its superiority as a therapy option, larger human-based clinical trials comparing magnesium to other available treatments are essential.

Although some studies suggest a beneficial relationship between magnesium and DN, data are insufficient to establish magnesium as a superior therapy option. More research is needed to fully understand its medicinal potential. The optimal dosage and duration of magnesium supplementation and its long-term effects as a therapeutic remain uncertain. Conducting well-designed trials is crucial to address these concerns and evaluate the feasibility and safety of incorporating magnesium into DN treatment.

## Conclusions

To summarize, the pathophysiology of DN is complex and multifaceted, and oxidative stress is important in its genesis and progression. Magnesium appears to be a promising therapeutic agent for reducing oxidative stress. In addition to its role in lowering oxidative stress, magnesium demonstrates various renoprotective mechanisms in the context of CKD to improve DN, such as anti-inflammatory qualities, antifibrotic properties, tubular integrity maintenance, vasoprotective effects, modulation of renal hemodynamics, and overall renal function preservation.

Clinical investigations have shown that magnesium supplementation positively affects CKD patients with DN. However, further well-designed clinical trials are needed to assess its true potential as an additional therapy for DN and to inform evidence-based clinical guidelines for its use. As research in this area progresses, a better knowledge of magnesium’s mechanisms of action and its appropriate role in the management of DN may lead to better patient outcomes and a higher quality of life for people suffering from this debilitating condition. Furthermore, research is needed to identify the therapeutic dose of magnesium and the long-term effects of magnesium supplementation as an additional therapy in the treatment of CKD.
